# Efficacy and safety of immune checkpoint inhibitors for EGFR mutated non-small cell lung cancer: a network meta-analysis

**DOI:** 10.3389/fimmu.2024.1512468

**Published:** 2024-12-23

**Authors:** Lin Zhu, Wenjuan He, Cunlei Xie, Yang Shu, Chunxia Zhang, Yawen Zhu

**Affiliations:** ^1^ Wuhan Wuchang Hospital, Wuchang Hospital Affiliated to Wuhan University of Science and Technology, Wuhan, China; ^2^ Department of Pharmacy, Wuhan Fourth Hospital, Wuhan, China

**Keywords:** treatment strategy, immunotherapy, overall survival, progression-free survival, adverse events

## Abstract

**Introduction:**

Non-small cell lung cancer (NSCLC) constitutes approximately 80–85% of cancer-related fatalities globally, and direct and indirect comparisons of various therapies for NSCLC are lacking. In this study, we aimed to compare the efficacy and safety of immune checkpoint inhibitors (ICIs) in patients with epidermal growth factor receptor (EGFR)-mutated NSCLC.

**Methods:**

The electronic databases were systematically searched from inception until March 18, 2024. Studies comparing two or more treatments involving ICIs in patients with EGFR-mutated NSCLC were included. The primary endpoints were overall survival (OS) and progression-free survival (PFS), and the secondary endpoints were overall response rate (ORR), any grade adverse events (AEs), grade ≥3 AEs, and AEs requiring treatment discontinuation. The R software with the gemtc package was used to compare the outcomes of the different treatments.

**Results:**

In 11 eligible studies involving 1462 patients and 5 regimens (chemotherapy [chemo], ICI, ICI+chemo, antiangiogenesis+chemo, and ICI+antiangiogenesis+chemo), ICI+antiangiogenesis+chemo achieved the most favorable OS compared to chemo (HR=0.74, 95% CI 0.41–1.23), ICI+chemo (HR=0.94, 95% CI 0.57–1.46), and ICI (HR=0.58, 95% CI 0.27–1.08) and a nearly equivalent effect to antiangiogenesis+chemo (HR=1.01, 95% CI 0.52–1.92). The PFS and ORR results were similar to those of OS. ICI monotherapy exhibited the lowest toxicity profile.

**Conclusions:**

These findings indicate that ICI+antiangiogenesis+chemo may be potentially beneficial for patients with EGFR-mutated NSCLC. However, the observed difference was not significant; thus, more studies are needed to confirm the efficacy and safety of the combined ICI treatment strategy.

**Systematic Review Registration:**

https://www.crd.york.ac.uk/PROSPERO/, identifier CRD42023424781.

## 1 Introduction

Lung cancer is the most prevalent type of cancer and the primary cause of cancer-related mortality globally (1, 2), with non-small cell lung cancer (NSCLC) accounting for approximately 80–85% of cases (3). Most NSCLCs are locally advanced or metastatic at diagnosis, reducing opportunities for surgery (4, 5), thereby resulting in a diminished overall 5-year relative survival rate and an unfavorable prognosis (6, 7).

Epidermal growth factor receptor (EGFR) mutations occur in many patients with NSCLC (8). Currently, EGFR tyrosine kinase inhibitors (TKI) are widely used clinically owing to their inhibitory effects on neovascularization, invasion, metastasis, and tumor cell growth (9, 10). Presently, three generations (gens) of EGFR-TKIs exist as follows: gefitinib, erlotinib and icotinib (1st gen), afatinib and dacomitinib (2nd gen), and osimertinib (3rd gen). However, most patients eventually experience disease progression and develop resistance within 9–12 months, limiting the long-term efficacy of EGFR-TKIs (11, 12).

In the last decade, immune checkpoint inhibitors (ICIs) targeting programmed death 1 (PD-1), programmed death ligand 1 (PD-L1), and cytotoxic T lymphocyte antigen 4 have dramatically changed the prognosis of patients with advanced NSCLC (13); however, their clinical benefits are constrained in individuals with EGFR-mutated NSCLC (14). KEYNOTE-001 indicated that the objective response rate (ORR), progression-free survival (PFS), and median overall survival (OS) were only 4%, 56 days, and 120 days, respectively, for 26 patients on pembrolizumab in a phase I study, and none of the patients had an objective response in subsequent phase II trials (15). CheckMate 012 also revealed lower ORR and PFS in patients with EGFR mutations than in those with wild-type mutations on first-line nivolumab monotherapy (ORR: 14% versus 30%; PFS: 1.8 versus 8.8 months) (16). In the ORIENT-31 study, Lu et al. (17) reported that sintilimab in combination with chemo significantly improved PFS compared to chemo alone (median PFS 5.5 months [95% CI 4.5–6.1] vs. 4.3 months [4.1–5.3]; hazard ratio [HR] 0.72 [95% CI 0.55–0.94]; two-sided p=0.016). These results demonstrate the potential benefit of ICIs in patients with EGFR-mutated NSCLC who had previously progressed on treatment with tyrosine kinase inhibitors. However, in a retrospective study, immunotherapy with platinum doublet chemo post-osimertinib was associated with a worse OS than platinum doublet chemo alone (18).

The efficacy and safety of ICIs remain controversial in patients with EGFR-mutated NSCLC, particularly in those with EGFR-TKI progression. Despite numerous ICI regimens for treating EGFR-mutated NSCLC, direct and indirect comparisons among these agents are lacking. Therefore, using a well-designed and comparative synthesis, we performed a systematic review and network meta-analysis (NMA) to directly and indirectly compare the advantages of these treatments and assess the efficacy and safety of ICIs in patients with EGFR-mutated NSCLC.

## 2 Materials and methods

### 2.1 Study selection

Two investigators independently screened the titles and abstracts to eliminate irrelevant articles and further screened dissertations by reading the full text. Disagreements were resolved through a group discussion.

The inclusion criteria were as follows:

- Studies that enrolled patients with histologically or cytologically confirmed NSCLC with EGFR mutations.
- Studies with reported outcomes of at least one of the following:

OS, defined as the time from randomization to death from any cause; PFS, defined as the time from randomization to the first disease progression (locoregional or distant) or all-cause mortality; ORR, defined as the rate at which patients achieve an objective response; toxicity, characterizing as adverse events (AEs) of any grade, grade 3 or higher (grade ≥3 AEs), or requiring treatment discontinuation.

- The study design included randomized controlled trials (RCTs) and real-world studies (RWSs).

The exclusion criteria were as follows:

- Conferences, abstracts, protocols, single-arm studies, nonhuman research, systematic reviews, and case reports.
- For studies based on the same trial, only the most recent trial was included.

We conducted this meta-analysis according to the preferred reporting items for systematic reviews and meta-analysis extension statements for NMA (19). This study protocol was registered in the Prospective Register of Systematic Reviews (PROSPERO CRD42023424781). Institutional Review Board exemption was granted due to the innocuousness of this review study.

Two investigators systematically searched PubMed, Web of Science, and Cochrane Library databases for relevant articles from inception to March 18, 2024, with no language limits, using a combination of the main search terms, including “ICI,” “NSCLC,” and “EGFR.” The reference lists of relevant articles were examined for additional articles, and the detailed search strategies are listed in **Supplementary Table S1**.

### 2.2 Data extraction and quality assessment

Extracted publication details included the first author’s name, year of publication, country, study design, phase of the trial, setting, diagnostic criteria, treatment regimens of the intervention and control groups, the number of participants in each arm, follow-up duration, patient characteristics (age and male ratio), primary clinical outcomes (OS and PFS), and secondary clinical outcomes (ORR, any grade AEs, grade ≥3 AEs, and AEs requiring treatment discontinuation). For primary clinical outcomes, we extracted the hazard ratios (HRs) and 95% confidence intervals (CIs) published in each study. When HRs could not be extracted directly, we used GetData software to capture data from Kaplan–Meier curves and calculated them using the digital computation chart developed by Tierney et al. (20). If the HRs and Kaplan–Meier curves could not be obtained, we extracted data using Cox univariate analysis. For secondary clinical outcomes, we directly extracted the corresponding number of cases from each study. The relative ratio (RR) and 95% CIs were used to evaluate the ORR and AEs, respectively. Data from six studies (17, 21–25) were extracted from original articles, whereas data from four studies (18, 26–28) were extracted from Kaplan–Meier curves. PFS data were extracted from original articles, and OS data were extracted from the Kaplan–Meier curves in Chen et al. (29).

The RWS quality was assessed using the Newcastle–Ottawa Scale (NOS), which comprises the following three major parameters: selection, comparability, and exposure or outcome. Scores >6 points indicate high-quality studies (30). RCTs were evaluated using the Cochrane risk of bias (ROB) Tool in Review Manager 5.3 software. Six aspects were evaluated as follows: random sequence generation, allocation concealment, blinding of participants and personnel or outcome assessment, incomplete outcome data, selective reporting, and other sources of bias. Each study was graded into low, high, or unclear (moderate) bias (31).

Two investigators independently extracted data and assessed the quality of the included studies. Discrepancies were resolved through consensus and arbitration within groups.

### 2.3 Statistical analysis

We synthesized evidence and compared the efficacy and safety. Efficacy was reported as PFS, OS, ORR, and safety was reported as any grade AEs, grade ≥3 AEs, and AEs requiring treatment discontinuation. Network plots were generated for the different outcomes of the regimens to illustrate the comparisons between different treatments in the included studies using Stata 14.0. We performed Bayesian NMA using the R software 4.3.2 (R Project for Statistical Computing; gemtc package) (32). For efficacy and safety outcomes, 20,000 sample iterations were generated with 5,000 burn-ins and a thinning interval of 1 (33). The two fundamental assumptions underlying the NMA are transitivity (the exchangeability across studies to compare two treatments via a third one) and consistency (the direct and indirect estimates are statistically similar) (34). Heterogeneity was assessed using the *Q* test and *I* ^2^ statistic within a visual forest plot, and the heterogeneity was considered low, moderate, and high when *I* ^2^ <25%, 25%≦*I* ^2^ <50%, and *I* ^2^≧50%, respectively (30). Inconsistency was calculated using the node splitting approach, where direct and indirect evidence were separately contrasted for a particular comparison (node). Moreover, for each outcome, we estimated the probability of each agent at each possible rank, and the surface under the cumulative ranking (SUCRA) curve was used to rank the safety and clinical outcomes of various regimens, with a higher SUCRA value indicating a better outcome ranking (35). A regimen with an HR <1 for OS and PFS or an RR >1 for ORR was deemed preferable, whereas an RR >1 for AEs indicated a greater likelihood of toxic effects. The risk of inconsistency was low (95% CI: 1). A funnel plot was constructed to further detect publication bias in the included studies, and significant asymmetry was defined as the presence of publication bias. Statistical significance was set at *p*<0.05.

## 3 Results

### 3.1 Systematic review and characteristics

**Figure 1:**
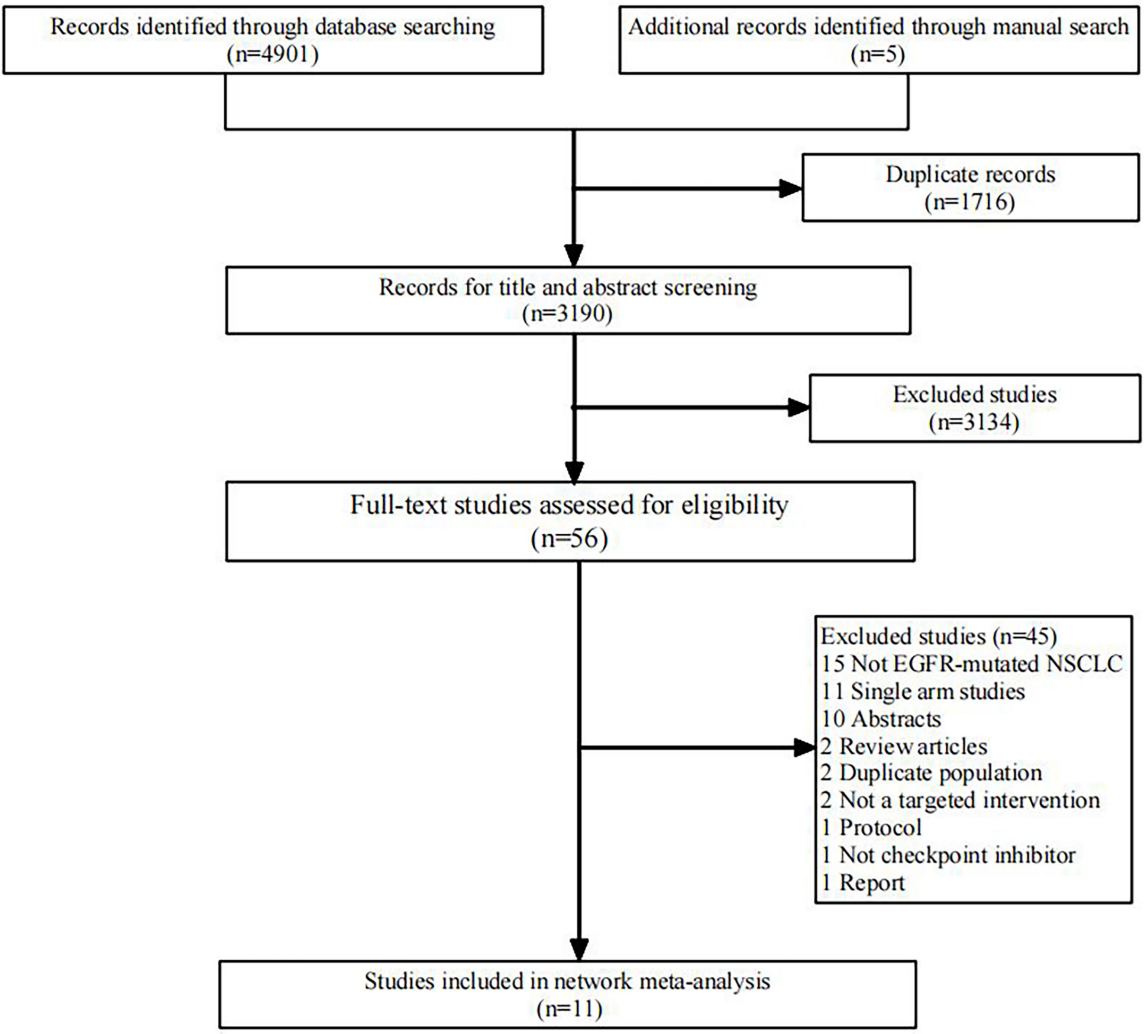
Flowchart of literature search and selection followed the preferred reporting items for systematic reviews and meta-analysis guidelines. EGFR, epidermal growth factor receptor; NSCLC, non-small cell lung cancer.

**Figure 2:**
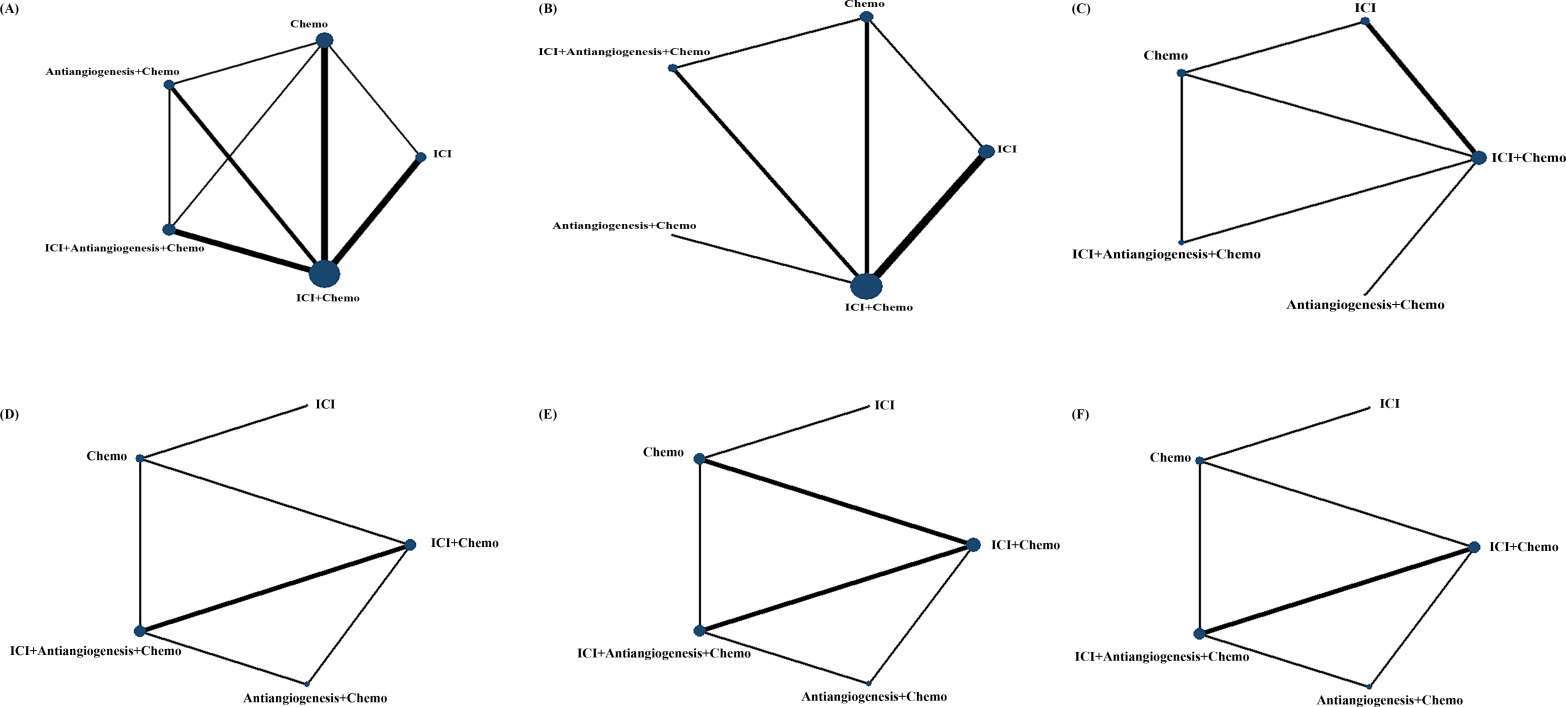
Comparative network plots for efficacy and safety of ICI for patients with EGFR-mutated NSCLC. Circular nodes represent the different types of treatments, while lines depict head-to-head comparison. The size of the node and the width of the line are proportional to the number of patients and comparisons, respectively. Comparisons were conducted using the Bayesian framework on **(A)** OS. **(B)** PFS. **(C)** ORR. **(D)** Safety assessed according to AEs of any grade. **(E)** Safety assessed according to grade ≥3 AEs. **(F)** Safety assessed according to AEs of any grade leading to treatment discontinuation occurred. AEs, adverse events; Chemo, chemotherapy; ICI, immune checkpoint inhibitor; EGFR, epidermal growth factor receptor; NSCLC, non-small cell lung cancer; OS, overall survival; PFS, progression-free survival; ORR, objective response rate.

We initially screened 4108 articles from the databases according to the search strategy, and 56 articles were retrieved and reviewed for their full text. Eventually, 11 articles met the inclusion criteria for this NMA, comprising two RCTs (17, 23) and nine RWSs (18, 21, 22, 24–29) with 1462 patients. **Figure 1** illustrates the process of the study selection process. These patients received the following five regimens: ICI+chemo, chemo, ICI, antiangiogenesis+chemo, and ICI+antiangiogenesis+chemo. ICIs included atezolizumab, nivolumab, pembrolizumab, and sintilimab. Chemo included carboplatin, paclitaxel, pemetrexed, cisplatin, and platinum. Antiangiogenesis included bevacizumab and its biosimilar agent (IBI305). The networks are presented in **Figure 2**, with nodes representing regimens and edges indicating RCTs or RWSs for pairs of treatments. All primary features are detailed in **Table 1**.

**Table 1:** Main characteristics of the studies included in the network meta-analysis.

Author	Year	Country	Study design, Phase	Setting	Diagnostic criteria	Treatment arm	Sample size (No.)	Age (Range)	Male ratio (%)	Follow-up (months)
Hayashi et al.	2022	Japan	prospective, randomized, II	37 sites of West Japan Oncology Group	locally advanced, metastatic, or recurrent non-squamous NSCLC positive for an activating mutation of EGFR	nivolumab	52	70.5 (51–84)	46.2	25.5 (0.1-46.1)
						carboplatin+pemetrexed	50	67 (45–83)	38	23.4 (1.6-48)
White et al.	2021	US	retrospective, NR	Stanford Cancer Institute and Massachusetts General Hospital	stage IV or recurrent metastatic NSCLC with a sensitizing EGFR mutation	chemotherapy+immunotherapy	12	56.5	50	NR
						chemotherapy	57	62.9	37	
						chemotherapy+immunotherapy	12	56.5	50	
						chemotherapy+bevacizumab	35	60.9	29	
Chen et al.	2022	China	retrospective, NR	Peking Union Medical College Hospital	NSCLC with sensitive EGFR mutations	chemotherapy+pembrolizumab	82	65.5 (32-82)	40.2	12.5
						chemotherapy	82	59 (36-80)	39.0	13.1
Nogami et al.	2021	Japan	randomized,open-label, III	240 study centers in 26 countries	chemotherapy-naive, metastatic, non-squamous NSCLC with EGFR mutations	atezolizumab+bevacizumab+carboplatin+paclitaxel	34	64.0 (37–76)	52.9	39.3
						atezolizumab+carboplatin+paclitaxel	45	63.0 (38–82)	37.8	
						bevacizumab+carboplatin+paclitaxel	44	61.5 (31–81)	45.5	
Lu et al.	2023	China	randomized, double-blind, III	52 centers across China	locally advanced or metastatic EGFR-mutated non-squamous NSCLC	sintilimab+IBI305+pemetrexed+cisplatin	158	58.5 (52.0–65.0)	41	12.9
						sintilimab+pemetrexed+cisplatin	158	57.5 (52.0–65.0)	41	15.1
						pemetrexed+cisplatin	160	56.0 (51.0–64.5)	40	14.4
Yu et al.	2021	China	retrospective, NR	Shanghai Pulmonary Hospital and Shanghai Chest Hospital	EGFR-TKI resistance in patients with EGFR-mutant advanced NSCLC	chemotherapy+immunotherapy	44	63.5 (19–76)	52.3	8.9
						chemotherapy+antiangiogenesis	100	58.5 (36–75)	45	
Kuo et al.	2019	China	retrospective, NR	Chang Gung Memorial Hospital	advanced or metastatic lung cancer who were administered at least one cycle of ICI treatment	immune checkpoint inhibitor+chemotherapy	5	NR	NR	NR
						immune checkpoint inhibitor	16			
Morimoto et al.	2022	Japan	retrospective, NR	12 institutions in Japan	histologically confirmed NSCLC, confirmed EGFR-activating mutation	immune checkpoint inhibitor	42	68 (43-85)	50.0	25.6
						immune checkpoint inhibitor+ chemotherapy	38	66 (39-79)	57.9	15.3
Shen et al.	2021	China	retrospective, observational, NR	a tertiary medical center	stage IV EGFR-mutant NSCLC	immune checkpoint inhibitor	22	65.5 (45-78)	45.5	16.76
						immune checkpoint inhibitor+ chemotherapy	8	67.5 (55-85)	37.5	
Bylicki et al.	2023	France	multicenter, open-label, non-randomized, II	27 centers	stage IIIB/IV non-squamous NSCLC patients with EGFR mutation or ALK/ROS1 fusion	platinum+ pemetrexed+ atezolizumab+ bevacizumab	62	NR	NR	14.8
						platinum+ pemetrexed+ atezolizumab	70			13.1
Chen et al.	2021	China	retrospective, NR	Shanghai Chest Hospital	stage IV NSCLC with positive EGFR mutation	Pembrolizumab	32	61 (39-80)	59.4	NR
						pembrolizumab+chemotherapy	26	66 (54-78)	50	
						pembrolizumab+anlotinib	28	59 (41-78)	57.1	

EGFR, epidermal growth factor receptor; NSCLC, non-small cell lung cancer; TKI, tyrosine kinase inhibitors; NR, not report.

### 3.2 NMA in EGFR-mutated NSCLC for efficacy

For OS (**Table 2A**; **Supplementary Figure S2A**), ICI+antiangiogenesis+chemo (HR=0.74, 95% CI 0.72–2.32), antiangiogenesis+chemo (HR=0.73, 95% CI 0.38–1.32), and ICI+chemo (HR=0.78, 95% CI 0.50–1.19) prolonged OS compared to chemo, albeit without significant difference, whereas ICI reduced OS compared to chemo (HR=1.26, 95% CI 0.72–2.32). No significant differences were observed between combination treatments.

**Table 2:** Pooled estimates of the network meta-analysis for patients with EGFR-mutated NSCLC.

A. Hazard ratios (HR) with 95% confidence interval (CI) for OS				
**Antiangiogenesis+Chemo**	1.38 (0.76, 2.65)	1.73 (0.85, 3.94)	1.01 (0.52, 1.92)	1.08 (0.61, 1.97)
0.73 (0.38, 1.32)	**Chemo**	1.26 (0.72, 2.32)	0.74 (0.41, 1.23)	0.78 (0.50, 1.19)
0.58 (0.25, 1.17)	0.80 (0.43, 1.38)	**ICI**	0.58 (0.27, 1.08)	0.62 (0.35, 1.02)
0.99 (0.52, 1.92)	1.36 (0.82, 2.45)	1.71 (0.92, 3.64)	**ICI+Antiangiogenesis+Chemo**	1.06 (0.68, 1.75)
0.93 (0.51, 1.64)	1.28 (0.84, 1.98)	1.61 (0.98, 2.83)	0.94 (0.57, 1.46)	**ICI+Chemo**
B. HR with 95% CI for PFS				
**Antiangiogenesis+Chemo**	1.18 (0.39, 3.47)	1.70 (0.59, 5.05)	0.65 (0.21, 2.04)	0.88 (0.34, 2.26)
0.84 (0.29, 2.56)	**Chemo**	1.44 (0.79, 2.76)	0.55 (0.28, 1.14)	0.74 (0.44, 1.28)
0.59 (0.20, 1.68)	0.70 (0.36, 1.26)	**ICI**	**0.38 (0.18, 0.82)**	**0.52 (0.31, 0.84)**
1.53 (0.49, 4.74)	1.82 (0.88, 3.63)	**2.61 (1.22, 5.69)**	**ICI+Antiangiogenesis+Chemo**	1.35 (0.71, 2.48)
1.14 (0.44, 2.95)	1.35 (0.78, 2.29)	**1.93 (1.20, 3.26)**	0.74 (0.40, 1.40)	**ICI+Chemo**
C. Relative risk (RR) with 95% CI for ORR				
**Antiangiogenesis+Chemo**	1.90 (0.19, 18.34)	0.48 (0.05, 4.45)	3.11 (0.28, 35.65)	2.25 (0.37, 13.51)
0.53 (0.05, 5.30)	**Chemo**	0.26 (0.05, 1.06)	1.64 (0.32, 8.54)	1.18 (0.29, 4.90)
2.06 (0.22, 21.61)	3.90 (0.94, 18.22)	**ICI**	6.41 (0.97, 47.19)	**4.63 (1.21, 20.90)**
0.32 (0.03, 3.62)	0.61 (0.12, 3.13)	0.16 (0.02, 1.03)	**ICI+Antiangiogenesis+Chemo**	0.72 (0.14, 3.75)
0.44 (0.07, 2.67)	0.85 (0.20, 3.40)	**0.22 (0.05, 0.83)**	1.39 (0.27, 7.14)	**ICI+Chemo**
D. RR with 95% CI for safety assessed according to any grade AEs				
**Antiangiogenesis+Chemo**	1.07 (0.68, 1.88)	0.81 (0.43, 1.67)	1.12 (0.79, 1.91)	1.00 (0.69, 1.49)
0.93 (0.53, 1.47)	**Chemo**	0.75 (0.47, 1.18)	1.04 (0.75, 1.56)	0.93 (0.62, 1.30)
1.24 (0.60, 2.35)	1.33 (0.85, 2.11)	**ICI**	1.40 (0.80, 2.59)	1.23 (0.67, 2.17)
0.89 (0.52, 1.26)	0.96 (0.64, 1.34)	0.71 (0.39, 1.25)	**ICI+Antiangiogenesis+Chemo**	0.89 (0.60, 1.14)
1.00 (0.67, 1.45)	1.07 (0.77, 1.61)	0.81 (0.46, 1.50)	1.13 (0.88, 1.66)	**ICI+Chemo**
E. RR with 95% CI for safety assessed according to grade ≥3 AEs				
**Antiangiogenesis+Chemo**	1.08 (0.68, 1.71)	0.85 (0.23, 2.98)	1.16 (0.78, 1.77)	0.96 (0.64, 1.48)
0.93 (0.58, 1.48)	**Chemo**	0.80 (0.24, 2.53)	1.08 (0.81, 1.46)	0.89 (0.70, 1.17)
1.17 (0.34, 4.26)	1.26 (0.40, 4.23)	**ICI**	1.36 (0.42, 4.72)	1.12 (0.35, 3.87)
0.86 (0.56, 1.29)	0.92 (0.69, 1.23)	0.73 (0.21, 2.40)	**ICI+Antiangiogenesis+Chemo**	0.83 (0.64, 1.08)
1.04 (0.68, 1.56)	1.12 (0.85, 1.43)	0.89 (0.26, 2.88)	1.21 (0.93, 1.56)	**ICI+Chemo**
F. RR with 95% CI for safety assessed according to AEs of any grade leading to treatment discontinuation occurred				
**Antiangiogenesis+Chemo**	0.81 (0.13, 4.68)	0.31 (0.02, 3.53)	2.35 (0.59, 9.53)	1.06 (0.24, 4.33)
1.24 (0.21, 7.80)	**Chemo**	0.38 (0.06, 2.13)	2.90 (0.82, 11.30)	1.31 (0.35, 5.01)
3.27 (0.28, 45.90)	2.61 (0.47, 17.80)	**ICI**	7.72 (0.92, 80.36)	3.46 (0.39, 35.13)
0.43 (0.10, 1.70)	0.34 (0.09, 1.21)	0.13 (0.01, 1.08)	**ICI+Antiangiogenesis+Chemo**	0.45 (0.16, 1.17)
0.95 (0.23, 4.10)	0.76 (0.20, 2.88)	0.29 (0.03, 2.55)	2.22 (0.86, 6.25)	**ICI+Chemo**

AEs, adverse events; Chemo, chemotherapy; ICI, immune checkpoint inhibitor; EGFR, epidermal growth factor receptor; NSCLC, non-small cell lung cancer; OS, overall survival; PFS, progression-free survival; ORR, objective response rate. Bold indicates different regimens, and colored represents significant differences.

The results of PFS (**Table 2B**; **Supplementary Figure S2B**) were similar to those of the OS. ICI+antiangiogenesis+chemo (HR=0.55, 95% CI 0.28–1.14), antiangiogenesis+chemo (HR=0.84, 95% CI 0.29–2.56), and ICI+chemo (HR=0.74, 95% CI 0.44–1.28) showed prolonged PFS compared to chemo, with no significant difference, whereas ICI reduced PFS compared to chemo (HR=1.44, 95% CI 0.79–2.76). ICI+antiangiogenesis+chemo yielded a better benefit in PFS than any other treatment (antiangiogenesis+chemo: HR=0.65, 95% CI, 0.21–2.04; ICI+chemo: HR=0.74, 95% CI 0.40–1.40).

For ORR (**Table 2C**; **Supplementary Figure S2C**), ICI+antiangiogenesis+chemo exhibited a tendency toward a higher ORR than chemo (HR=1.64, 95% CI 0.32–8.54) and any other treatment (antiangiogenesis+chemo: HR=3.11, 95% CI 0.28–35.65; ICI+chemo: HR=1.39, 95% CI 0.27–7.14; ICI: HR=6.41, 95% CI 0.97–47.19).

### 3.3 NMA in EGFR-mutated NSCLC for safety

For any grade AEs (**Table 2D**; **Supplementary Figure S2D**), each point estimates of the combined RRs exceeded 1 in ICI+antiangiogenesis+chemo treatment, indicating that ICI+antiangiogenesis+chemo may increase the incidence more than any other treatment (antiangiogenesis+chemo: HR=1.12, 95% CI 0.79–1.91; ICI+chemo: HR=1.13, 95% CI 0.88–1.66; chemo: HR=1.04, 95% CI 0.75–1.56; and ICI: HR=1.40, 95% CI 0.80–2.59). In contrast, all point estimates of the pooled RRs were lower than 1 in the ICI treatment, indicating that ICI yielded the lowest incidence compared to any other treatment (ICI+antiangiogenesis+chemo: HR=0.71, 95% CI 0.39–1.25; antiangiogenesis+chemo: HR=0.81, 95% CI 0.43–1.67; ICI+chemo: HR=0.81, 95% CI 0.46–1.50; and chemo: HR=0.75, 95% CI 0.47–1.18).

Regarding grade ≥3 AEs (**Table 2E**; **Supplementary Figure S2E**) and AEs leading to treatment discontinuation (**Table 2F**; **Supplementary Figure S2F**), the results were similar to those of any grade AEs.

### 3.4 Rank probabilities

**Figure 3:**
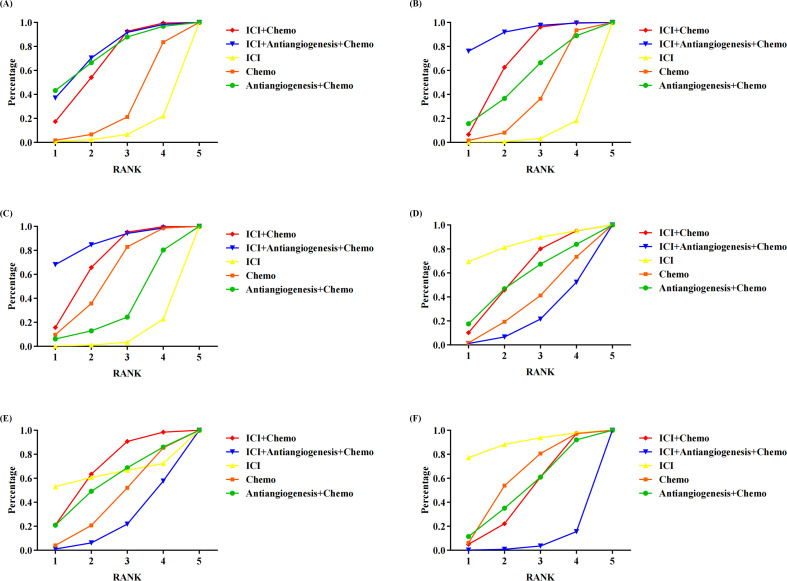
Bayesian ranking profiles assessing the efficacy and safety of ICI for patients with EGFR-mutated NSCLC. The profiles indicate the probability of each treatment being ranked from first to last on **(A)** OS. **(B)** PFS. **(C)** ORR. **(D)** safety assessed according to any grade AEs. **(E)** Safety assessed according to grade ≥3 AEs. **(F)** Safety assessed according to AEs of any grade leading to treatment discontinuation occurred. Different colored lines represent different interventions. The position of each line on the graph corresponds to the ranking probability of each intervention. AEs, adverse events; Chemo, chemotherapy; ICI, immune checkpoint inhibitor; EGFR, epidermal growth factor receptor; NSCLC, non-small cell lung cancer; OS, overall survival; PFS, progression-free survival; ORR, objective response rateTables.

**Figure 3** shows the Bayesian ranking profiles of various comparable treatments. Among EGFR-mutated NSCLC, ICI+antiangiogenesis+chemo was most likely to be ranked first for OS (74%), PFS (92%), ORR (87%), ICI for any grade AEs (84%) and any grade AEs leading to treatment discontinuation (89%), and ICI+chemo for grade ≥3 AEs (68%).

### 3.5 Quality assessment

During the literature quality assessment, all RWSs were assessed as high quality with NOS scores >6 points. However, one study (**Supplementary Table S2**) was evaluated as low risk, whereas two were classified as moderate risk using the ROB tool owing to concerns regarding blinding (**Supplementary Figure S1**).

### 3.6 Heterogeneity and inconsistency assessment

Forest plots with heterogeneity estimates are shown in **Supplementary Figure S3**. These results suggest low or moderate heterogeneity across most of the outcomes. An analysis of inconsistency among the direct, indirect, and overall effects showed low inconsistency with *p*>0.05 (**Supplementary Figure S4**). The funnel plot for all outcomes was almost symmetric, confirming the absence of publication bias (**Supplementary Figure S5**).

## 4 Discussion

This NMA included 11 articles, comprising two RCTs and nine RWSs, involving 1462 patients and evaluating five regimens. It summarized the comparative efficacy and safety of ICIs and combination therapies for patients with EGFR-mutated NSCLC using R software with the gemtc package. The results of this study indicated that ICI+antiangiogenesis+chemo achieved greater survival benefits than the other treatments regarding OS, PFS, and ORR. However, it was associated with a higher incidence of AEs, although this difference was not significant.

EGFR-TKIs are recommended as the standard first-line treatment for patients with advanced EGFR-mutated NSCLC (36). However, long-term EGFR-TKI resistance is inevitable. Currently, the main indication for first-line therapy with ICIs is in patients with wild-type EGFR because the PD-L1 expression level in EGFR mutations is lower than that in the wild-type (37–39). Tumor cells often exhibit high PD-L1 expression under the influence of various cytokines. When T cells recognize tumor cells, PD-L1 on the tumor cell surface binds to PD-1 on T cells, thereby inhibiting T cell proliferation and their cytotoxic effects on tumor cells, leading to immune evasion by the tumor (40, 41). ICIs block the interaction between PD-1 and PD-L1, thereby restoring the antitumor activity of T cells. Consequently, PD-L1 expression is currently the most widely used ICI predictive marker. This is not only because ICIs target PD-1 receptor-ligand interactions but also because PD-L1 expression correlates with parameters associated with immune activation in the tumor, such as activated CD8^+^ T cells and antigen presentation. Therefore, patients who are PD-L1 negative or have low expression are more prone to developing resistance to ICIs (42). Kuo et al. (25) conducted a study comparing the efficacy of ICI combined with chemo versus chemo alone in EGFR-mutated NSCLC with PD-L1 expression levels of <50% and ≥50%. The results indicated that patients in the ICI plus chemo group experienced improved PFS compared to those receiving chemo alone. Notably, the lower the PD-L1 expression level, the greater the improvement observed (for PD-L1 TPS≥50%, PFS: ICI+Chemo vs Chemo HR=0.93, 95% CI 0.37-2.36; for PD-L1 TPS<50%, PFS: ICI+Chemo vs Chemo HR=0.86, 95% CI 0.39-1.92). In contrast, Hayashi et al. (21) compared the efficacy of ICI and chemo in EGFR-mutated NSCLC with PD-L1 TPS between 1% and 49% and PD-L1≥50%. Their findings showed that patients in the ICI group had improved PFS compared to those in the chemo group, with the benefit being more pronounced at higher PD-L1 expression levels (for 1%≤ PD-L1 TPS ≤ 49%, PFS: ICI vs Chemo HR=2.10, 95% CI 0.83-5.29; for PD-L1 TPS≥50%, PFS: ICI+Chemo vs Chemo HR=1.49, 95% CI 0.31-7.24). These findings suggest the need for further research to explore the relationship between PD-L1 expression levels and the efficacy of ICI in EGFR-mutated NSCLC.

EGFR mutation may reduce CD8^+^ T cell infiltration by activating transforming growth factor-β (TGFβ), leading to immunosuppression and lymphocyte depletion within the tumor (43). Additionally, under TGFβ induction, stromal cells can form a physical muscle fiber barrier around tumor cells, preventing T cell infiltration and migration (44). Patients with EGFR-mutated NSCLC and high CD73 expression can hydrolyze ATP into adenosine, exerting immunosuppressive effects by acting on A2a/A2b receptors. It can activate regulatory T cells and myeloid-derived suppressor cells, weaken the anti-tumor functions of dendritic and natural killer cells, polarize macrophages towards the M2 phenotype, and suppress T cell-mediated anti-tumor responses, thereby mediating the immune escape of tumors (45–47). The lack of effective tumor-killing effector cells in the tumor microenvironment of EGFR-mutated NSCLC and the dysfunction of effector cells are potential causes of poor immunotherapy outcomes in patients with EGFR-mutated NSCLC.

Vascular endothelial growth factor (VEGF) is a key factor in fostering angiogenesis and tumor growth (48). However, this neovascularization is structurally disorganized and dysfunctional, lacks pericellular and basement membrane wrapping, and has loose connections with the endothelium, resulting in reduced infiltration of cytotoxic T cells (49). Studies have shown that VEGF inhibitors can “normalize” tumor blood vessels, increase pericyte coverage, improve tumor vessel perfusion, and destroy the physical and chemical barriers of endothelial cells, resulting in an increased inflow of CD4^+^ and CD8^+^ T cells into the tumor parenchyma (50). Therefore, antiangiogenesis therapy can improve VEGF-induced tumor vascular system dysfunction, promote effector cell infiltration, and eliminate obstacles in tumor immunotherapy. ICIs induce CD4^+/^CD8^+^ T cells to produce interferonγ, increase lymphocyte infiltration and activation, promote tumor vascular normalization, and produce synergistic effects (51).

White et al. (18), Chen et al. (22), and Lu et al. (17) all compared the effects of ICI+chemo versus chemo alone on OS in EGFR-mutated NSCLC. The results indicated that, except for White’s study, all showed that ICI+chemo could improve OS in patients with EGFR-mutated NSCLC compared to chemo alone. There are two possible reasons for this: 1. In Lu’s study, the investigational drugs included 200 mg sintilimab, 15 mg/kg IBI305, 500 mg/m² pemetrexed, and 75 mg/m² cisplatin; in Chen’s study, the investigational drugs included pembrolizumab and platinum-based doublet chemotherapy; in White’s study, 54 patients received carboplatin/pemetrexed; 1 received carboplatin/paclitaxel; 1 received carboplatin/albumin-bound paclitaxel; 1 received carboplatin/gemcitabine, 12 patients received chemotherapy plus immunotherapy (carboplatin/pemetrexed/pembrolizumab), and 35 patients received chemotherapy plus bevacizumab (carboplatin/pemetrexed/bevacizumab). White’s study involved a wider variety of chemotherapy drugs, with significant differences between the different chemotherapy regimens. 2. It is possible that in White’s study, the HR was derived from points taken on the Kaplan-Meier curve, which may have introduced some errors. While, Chen and Lu both confirmed that ICI+chemo could improve PFS in patients with EGFR-mutated NSCLC compared to chemo alone.

Recently, a network meta-analysis on the efficacy and safety of ICIs for individuals with advanced EGFR-mutated NSCLC who progressed on EGFR tyrosine kinase inhibitors was published in *Lancet Oncology* (52). Our study differs from the recent study in *Lancet Oncology*. To clarify the study population, we focused specifically on EGFR-mutant NSCLC, excluding metastatic nonsquamous EGFR-mutant NSCLC. In terms of OS, PFS, and ORR, our conclusions align with those of the Lancet Oncology study. We found that ICI+antiangiogenesis+chemotherapy yielded the best OS, PFS, and ORR compared to any other treatment. However, due to the limited number of original studies in our analysis, we did not observe significant differences between ICI+antiangiogenesis+chemo and other treatment strategies, except for the benefit of ICI+antiangiogenesis+chemo over ICI alone in terms of PFS. In contrast, Zhao et al. (52) demonstrated significant differences between ICI+antiangiogenesis+chemo and other treatment strategies for both PFS and ORR, based on a larger number of original studies. Regarding safety, both studies found that ICI+antiangiogenesis+chemo was associated with a higher risk of any-grade adverse events compared to ICI+chemo and chemo alone.

This NMA has some limitations. First, the number of studies included was limited. Therefore, this study lacked a subgroup analysis based on smoking status, sex, or other associated factors, which might compromise the credibility and veracity of this assessment. Therefore, future studies should investigate these clinical characteristics using NMA. Second, variations in mechanisms and toxicities among ICIs (e.g., atezolizumab, nivolumab, pembrolizumab, and sintilimab), chemo drugs (carboplatin, paclitaxel, pemetrexed, cisplatin, and platinum), and anti-angiogenic drugs (bevacizumab, IBI305) incorporated into treatment regimens introduce heterogeneity. Third, data extraction from several studies in this NMA involved digitizing Kaplan–Meier curves from clinical trials rather than being based on exact PFS and OS for each patient. This approach may have resulted in minor deviations in our results.

To conclude, based on our results, it is inferred that combination therapy of ICI, antiangiogenesis, and chemotherapy holds potential benefits for patients with EGFR-mutated NSCLC, although without significant differences. Further studies are warranted to validate the efficacy and safety of combined ICI treatments.

## Data Availability

The original contributions presented in the study are included in the article/**Supplementary Material**. Further inquiries can be directed to the corresponding authors.
