# Supplementary material for: Efficacy and safety of immune checkpoint inhibitors for EGFR mutated non-small cell lung cancer: a network meta-analysis
Source: Front Immunol. 2024 Dec 23;15:1512468. doi: 10.3389/fimmu.2024.1512468 (PMC11701139; doi:10.3389/fimmu.2024.1512468)
Supplement: Supplementary file 1 [file Table1.docx]

**Supplementary table S1.** Search strategy in selective databases.

| Database | Search Term | Results |
| --- | --- | --- |
| PubMed# | #1: (immune checkpoint[Title/Abstract]) OR (immune therapy[Title/Abstract]) OR (immunotherapy[Title/Abstract]) OR (ipilimumab[Title/Abstract]) OR (tremelimumab[Title/Abstract]) OR (nivolumab[Title/Abstract]) OR (pembrolizumab[Title/Abstract]) OR (atezolizumab[Title/Abstract]) OR (durvalumab[Title/Abstract]) OR (avelumab[Title/Abstract]) OR (cemiplimab[Title/Abstract]) OR (toripalimab[Title/Abstract]) OR (camrelizumab[Title/Abstract]) OR (tislelizumab[Title/Abstract]) OR (BMS-936559[Title/Abstract]) OR (PD-1[Title/Abstract]) OR (PD 1[Title/Abstract]) OR (anti-PD-1[Title/Abstract]) OR (PD-L1[Title/Abstract]) OR (PD L1[Title/Abstract]) OR (anti-PD-L1[Title/Abstract]) OR (CTLA-4[Title/Abstract]) OR (CTLA 4[Title/Abstract]) OR (anti-CTLA-4[Title/Abstract]) OR (programmed cell death protein-1[Title/Abstract]) OR (programmed cell death protein[Title/Abstract]) OR (programmed cell death 1 receptor[Title/Abstract]) OR (programmed death-ligand 1 inhibitors[Title/Abstract]) OR (programmed death ligand 1 inhibitors[Title/Abstract]) OR (anti-cytotoxic T-lymphocyte antigen-4[Title/Abstract]) OR (cytotoxic T-lymphocyte associated antigen-4[Title/Abstract]) OR (cytotoxic T-lymphocyte-associated protein 4 inhibitors[Title/Abstract]) OR (cytotoxic T lymphocyte associated protein 4 inhibitors[Title/Abstract]) OR (cytotoxic T-lymphocyte-associated protein 4 inhibitor[Title/Abstract]) OR (cytotoxic T lymphocyte associated protein 4 inhibitor[Title/Abstract]) | 190,156 |
|  | #2: (non-small-cell lung cancer[Title/Abstract]) OR (non-small cell lung cancer[Title/Abstract]) OR (non small-cell lung cancer[Title/Abstract]) OR (non small cell lung cancer[Title/Abstract]) OR (non-small-cell lung carcinoma[Title/Abstract]) OR (non-small cell lung carcinoma[Title/Abstract]) OR (non small-cell lung carcinoma[Title/Abstract]) OR (non small cell lung carcinoma[Title/Abstract]) OR (NSCLC[Title/Abstract]) | 92,772 |
|  | #3: (epidermal growth factor receptor[Title/Abstract]) OR (EGFR[Title/Abstract]) | 108,286 |
|  | #4: #1 AND #2 AND #3 | 1,863 |
| Web of Science | #1: TI=(immune checkpoint OR immune therapy OR immunotherapy OR ipilimumab OR tremelimumab OR nivolumab OR pembrolizumab OR atezolizumab OR durvalumab OR avelumab OR cemiplimab OR toripalimab OR camrelizumab OR tislelizumab OR BMS-936559 OR PD-1 OR PD 1 OR anti-PD-1 OR PD-L1 OR PD L1 OR anti-PD-L1 OR CTLA-4 OR CTLA 4 OR anti-CTLA-4 OR programmed cell death protein-1 OR programmed cell death protein OR programmed cell death 1 receptor OR programmed death-ligand 1 inhibitors OR programmed death ligand 1 inhibitors OR anti-cytotoxic T-lymphocyte antigen-4 OR cytotoxic T-lymphocyte associated antigen-4 OR cytotoxic T-lymphocyte-associated protein 4 inhibitors OR cytotoxic T lymphocyte associated protein 4 inhibitors OR cytotoxic T-lymphocyte-associated protein 4 inhibitor OR cytotoxic T lymphocyte associated protein 4 inhibitor) | 140,249 |
|  | #2: TI=(non-small-cell lung cancer OR non-small cell lung cancer OR non small-cell lung cancer OR non small cell lung cancer OR non-small-cell lung carcinoma OR non-small cell lung carcinoma OR non small-cell lung carcinoma OR non small cell lung carcinoma OR NSCLC) | 96,941 |
|  | #3: TI=(epidermal growth factor receptor OR EGFR) | 49,518 |
|  | #4: AB=(immune checkpoint OR immune therapy OR immunotherapy OR ipilimumab OR tremelimumab OR nivolumab OR pembrolizumab OR atezolizumab OR durvalumab OR avelumab OR cemiplimab OR toripalimab OR camrelizumab OR tislelizumab OR BMS-936559 OR PD-1 OR PD 1 OR anti-PD-1 OR PD-L1 OR PD L1 OR anti-PD-L1 OR CTLA-4 OR CTLA 4 OR anti-CTLA-4 OR programmed cell death protein-1 OR programmed cell death protein OR programmed cell death 1 receptor OR programmed death-ligand 1 inhibitors OR programmed death ligand 1 inhibitors OR anti-cytotoxic T-lymphocyte antigen-4 OR cytotoxic T-lymphocyte associated antigen-4 OR cytotoxic T-lymphocyte-associated protein 4 inhibitors OR cytotoxic T lymphocyte associated protein 4 inhibitors OR cytotoxic T-lymphocyte-associated protein 4 inhibitor OR cytotoxic T lymphocyte associated protein 4 inhibitor) | 364,345 |
|  | #5: AB=(non-small-cell lung cancer OR non-small cell lung cancer OR non small-cell lung cancer OR non small cell lung cancer OR non-small-cell lung carcinoma OR non-small cell lung carcinoma OR non small-cell lung carcinoma OR non small cell lung carcinoma OR NSCLC) | 80,709 |
|  | #6: AB=(epidermal growth factor receptor OR EGFR) | 106,272 |
|  | #7: #1 AND #2 AND #3 | 426 |
|  | #8: #4 AND #5 AND #6 | 1,786 |
|  | #9: #7 OR #8 | 2,018 |
| Cochrane library | #1: (immune checkpoint OR immune therapy OR immunotherapy OR ipilimumab OR tremelimumab OR nivolumab OR pembrolizumab OR atezolizumab OR durvalumab OR avelumab OR cemiplimab OR toripalimab OR camrelizumab OR tislelizumab OR BMS-936559 OR PD-1 OR PD 1 OR anti-PD-1 OR PD-L1 OR PD L1 OR anti-PD-L1 OR CTLA-4 OR CTLA 4 OR anti-CTLA-4  OR programmed cell death protein-1 OR programmed cell death protein OR programmed cell death 1 receptor OR programmed death-ligand 1 inhibitors OR programmed death ligand 1 inhibitors OR anti-cytotoxic T-lymphocyte antigen-4 OR cytotoxic T-lymphocyte associated antigen-4 OR cytotoxic T-lymphocyte-associated protein 4 inhibitors OR cytotoxic T lymphocyte associated protein 4 inhibitors OR cytotoxic T-lymphocyte-associated protein 4 inhibitor OR cytotoxic T lymphocyte associated protein 4 inhibitor):ti | 14,913 |
|  | #2: (non-small-cell lung cancer OR non-small cell lung cancer OR non small-cell lung cancer OR non small cell lung cancer OR non-small-cell lung carcinoma OR non-small cell lung carcinoma OR non small-cell lung carcinoma OR non small cell lung carcinoma OR NSCLC):ti | 13,069 |
|  | #3: (epidermal growth factor receptor OR EGFR):ti | 2,705 |
|  | #4: #1 AND #2 AND #3 | 58 |
|  | #5: (immune checkpoint OR immune therapy OR immunotherapy OR ipilimumab OR tremelimumab OR nivolumab OR pembrolizumab OR atezolizumab OR durvalumab OR avelumab OR cemiplimab OR toripalimab OR camrelizumab OR tislelizumab OR BMS-936559 OR PD-1 OR PD 1 OR anti-PD-1 OR PD-L1 OR PD L1 OR anti-PD-L1 OR CTLA-4 OR CTLA 4 OR anti-CTLA-4  OR programmed cell death protein-1 OR programmed cell death protein OR programmed cell death 1 receptor OR programmed death-ligand 1 inhibitors OR programmed death ligand 1 inhibitors OR anti-cytotoxic T-lymphocyte antigen-4 OR cytotoxic T-lymphocyte associated antigen-4 OR cytotoxic T-lymphocyte-associated protein 4 inhibitors OR cytotoxic T lymphocyte associated protein 4 inhibitors OR cytotoxic T-lymphocyte-associated protein 4 inhibitor OR cytotoxic T lymphocyte associated protein 4 inhibitor):ab | 39,285 |
|  | #6: (non-small-cell lung cancer OR non-small cell lung cancer OR non small-cell lung cancer OR non small cell lung cancer OR non-small-cell lung carcinoma OR non-small cell lung carcinoma OR non small-cell lung carcinoma OR non small cell lung carcinoma OR NSCLC):ab | 14,195 |
|  | #7: (epidermal growth factor receptor OR EGFR):ab | 14,716 |
|  | #8: #5 AND #6 AND #7 | 924 |
|  | #9: #4 OR #8 | 930 |
| Time | 2024.3.18 | |
| Language restriction | No restriction | |
