# Supplementary material for: Efficacy and safety of immune checkpoint inhibitors for EGFR mutated non-small cell lung cancer: a network meta-analysis
Source: Front Immunol. 2024 Dec 23;15:1512468. doi: 10.3389/fimmu.2024.1512468 (PMC11701139; doi:10.3389/fimmu.2024.1512468)
Supplement: Supplementary file 2 [file Table2.docx]

| **First author,**  **publication year**  **(reference)** | **Selection** | | | | **Comparability** | **Outcome** | | | **Total score** |
| --- | --- | --- | --- | --- | --- | --- | --- | --- | --- |
|  | **Representativeness of the exposed cohort** | **Selection of the unexposed**  **cohort** | **Ascertainment**  **of exposure** | **Outcome of interest not present at start of study** | **Comparability of cohorts on the basis of the design or analysis** | **Assessment of outcome** | **Follow-up**  **long enough for outcomes**  **to occur ^#^** | **Adequacy of**  **follow-up**  **of cohorts §** |  |
| Yu, 2021 | ⚝ | ⚝ | ⚝ | ⚝ | ⚝⚝ | ⚝ | **-** | ⚝ | 8 |
| Kuo, 2019 | ⚝ | ⚝ | ⚝ | ⚝ | ⚝⚝ | ⚝ | - | - | 7 |
| Morimoto, 2022 | ⚝ | ⚝ | ⚝ | ⚝ | ⚝⚝ | ⚝ | - | - | 7 |
| Shen, 2021 | ⚝ | ⚝ | ⚝ | ⚝ | ⚝⚝ | ⚝ | **-** | ⚝ | 8 |
| Bylicki, 2023 | ⚝ | ⚝ | ⚝ | ⚝ | ⚝⚝ | ⚝ | **-** | ⚝ | 8 |
| Chen, 2021 | ⚝ | ⚝ | ⚝ | ⚝ | ⚝⚝ | ⚝ | - | - | 7 |
| White, 2021 | ⚝ | ⚝ | ⚝ | ⚝ | ⚝⚝ | ⚝ | - | - | 7 |
| Chen, 2022 | ⚝ | ⚝ | ⚝ | ⚝ | ⚝⚝ | ⚝ | **-** | ⚝ | 8 |

**Supplement Table S2.** Quality assessment of retrospective studies included in the network meta-analysis. *

*A study can be awarded a maximum of one star for each numbered item within the Selection and Outcome categories. A maximum of two stars can be given for Comparability.

**^#^**A cohort study with a follow-up time >5 y was assigned one star.

**^§^**A cohort study with a follow-up rate >90% was assigned one star.
