# Supplementary material for: Efficacy and safety of immune checkpoint inhibitors for EGFR mutated non-small cell lung cancer: a network meta-analysis
Source: Front Immunol. 2024 Dec 23;15:1512468. doi: 10.3389/fimmu.2024.1512468 (PMC11701139; doi:10.3389/fimmu.2024.1512468)
Supplement: Supplementary file 3 [file Table3.docx]

**Supplement Table S3.** Primary outcome of the studies included in the network meta-analysis.

| **Author** | **Intervention arm** | **Control arm** | **Outcomes** | **HR** | **95% CI** |
| --- | --- | --- | --- | --- | --- |
| Hayashi et al. | ICI | Chemo | OS | 0.88 | 0.53-1.47 |
|  |  |  | PFS | 1.92 | 1.27-2.90 |
| White et al. | ICI+ Chemo | Chemo | OS | 1.38 | 0.66-2.90 |
|  | Antiangiogenesis+ Chemo | Chemo | OS | 0.85 | 0.50-1.43 |
| Chen et al. | ICI+ Chemo | Chemo | OS | 0.49 | 0.31-0.75 |
|  |  |  | PFS | 0.64 | 0.46-0.89 |
| Nogami et al. | ICI+Antiangiogenesis+Chemo | Antiangiogenesis+Chemo | OS | 0.91 | 0.53-1.59 |
|  | ICI+Chemo | Antiangiogenesis+Chemo | OS | 1.16 | 0.71-1.89 |
| Lu et al. | ICI+Antiangiogenesis+Chemo | Chemo | OS | 0.98 | 0.72-1.34 |
|  | ICI+Chemo | Chemo | OS | 0.97 | 0.71-1.32 |
|  | ICI+Antiangiogenesis+Chemo | Chemo | PFS | 0.51 | 0.39-0.67 |
|  | ICI+Chemo | Chemo | PFS | 0.72 | 0.55-0.94 |
| Yu et al. | ICI+Chemo | Antiangiogenesis+Chemo | PFS | 0.875 | 0.565-1.355 |
|  |  |  |  |  |  |
| Kuo et al. | ICI+Chemo | ICI | PFS | 0.22 | 0.05-0.98 |
|  |  |  |  |  |  |
| Morimoto et al. | ICI+Chemo | ICI | OS | 0.62 | 0.36-1.06 |
|  |  |  | PFS | 0.89 | 0.56-1.41 |
| Shen et al. | ICI+Chemo | ICI | OS | 0.48 | 0.15-1.47 |
|  |  |  | PFS | 0.79 | 0.33-1.86 |
| Bylicki et al. | ICI+Antiangiogenesis+Chemo | ICI+Chemo | OS | 0.74 | 0.42-1.29 |
|  |  |  | PFS | 0.84 | 0.54-1.32 |
| Chen et al. | ICI+Chemo | ICI | OS | 0.34 | 0.13-0.85 |
|  |  |  | PFS | 0.37 | 0.20-0.70 |

Abbreviations: ICI: immune checkpoint inhibitor; Chemo: chemotherapy; OS: overall survival; PFS: progression-free survival; HR: hazard ratios; CI: confidence interval.
