# Supplementary material for: Efficacy and safety of immune checkpoint inhibitors for EGFR mutated non-small cell lung cancer: a network meta-analysis
Source: Front Immunol. 2024 Dec 23;15:1512468. doi: 10.3389/fimmu.2024.1512468 (PMC11701139; doi:10.3389/fimmu.2024.1512468)
Supplement: Supplementary file 4 [file Table4.docx]

**Supplement Table S4.** Secondary outcome of the studies included in the network meta-analysis.

| **Author** | **Intervention arm** | **ORR** |
| --- | --- | --- |
|  |  |  |
| Hayashi et al. | ICI | 5/52 (9.6%) |
|  | Chemo | 18/50 (36.0%) |
| Chen et al. | ICI+Chemo | NR |
|  | Chemo |  |
| Nogami et al. | ICI+antiangiogenesis+Chemo | NR |
|  | ICI+Chemo |  |
|  | antiangiogenesis+Chemo |  |
| Lu et al. | ICI+antiangiogenesis+Chemo | 76/158 (48.1%) |
|  | ICI+Chemo | 55/158 (34.8%) |
|  | Chemo | 47/160 (29.4%) |
| Yu et al. | ICI+Chemo | 13/44 (29.5%) |
|  | antiangiogenesis+Chemo | 13/100 (13.0%) |
| Shen et al. | ICI+Chemo | 2/8 (25%) |
|  | ICI | 2/22 (9.1%) |
| Chen et al. | ICI+Chemo | 6/26 (23.1%) |
|  | ICI | 1/32 (3.1%) |

Abbreviations: Chemo: chemotherapy; ICI: immune checkpoint inhibitor; ORR: objective response rate; NR: not report.
