# Supplementary material for: Efficacy and safety of immune checkpoint inhibitors for EGFR mutated non-small cell lung cancer: a network meta-analysis
Source: Front Immunol. 2024 Dec 23;15:1512468. doi: 10.3389/fimmu.2024.1512468 (PMC11701139; doi:10.3389/fimmu.2024.1512468)
Supplement: Supplementary file 5 [file Table5.docx]

**Supplement Table S5.** The adverse events data of included studies.

| **Author** | **Intervention arm** | **any grade AE, n (%)** | **Grade≥3 AEs, n (%)** | **AEs requiring treatment discontinuation, n (%)** |
| --- | --- | --- | --- | --- |
|  |  | **Responders/Sample size (%)** | | |
| Hayashi et al. | ICI | 32/52 (60.8%) | 5/52 (9.8%) | 3/52 (5.8%) |
|  | Chemo | 41/50 (82%) | 6/50 (12.0%) | 7/50 (14.0%) |
| Chen et al. | ICI+Chemo | NR | 37/82 (45.12%) | NR |
|  | Chemo |  | 35/82 (42.68%) |  |
| Nogami et al. | ICI+antiangiogenesis+Chemo | 33/33 (100%) | 22/33 (66.7%) | 14/33 (42.4%) |
|  | ICI+Chemo | 39/44 (88.6%) | 25/44 (56.8%) | 6/44 (13.6%) |
|  | antiangiogenesis+Chemo | 41/43 (95.3%) | 25/43 (58.1%) | 7/43 (16.3%) |
| Lu et al. | ICI+antiangiogenesis+Chemo | 157/158 (99.4%) | 94/158 (59.5%) | 28/158 (17.7%) |
|  | ICI+Chemo | 151/156 (96.8%) | 72/156 (46.2%) | 16/156 (10.3%） |
|  | Chemo | 159/160 (99.4%) | 91/160 (56.9%) | 11/160 (6.9%) |

Abbreviations: Chemo: chemotherapy; ICI: immune checkpoint inhibitor; AE: adverse event; NR: not report.
