# Supplementary figures and images for: Efficacy and safety of immune checkpoint inhibitors for EGFR mutated non-small cell lung cancer: a network meta-analysis
Source: Front Immunol. 2024 Dec 23;15:1512468. doi: 10.3389/fimmu.2024.1512468 (PMC11701139; doi:10.3389/fimmu.2024.1512468)

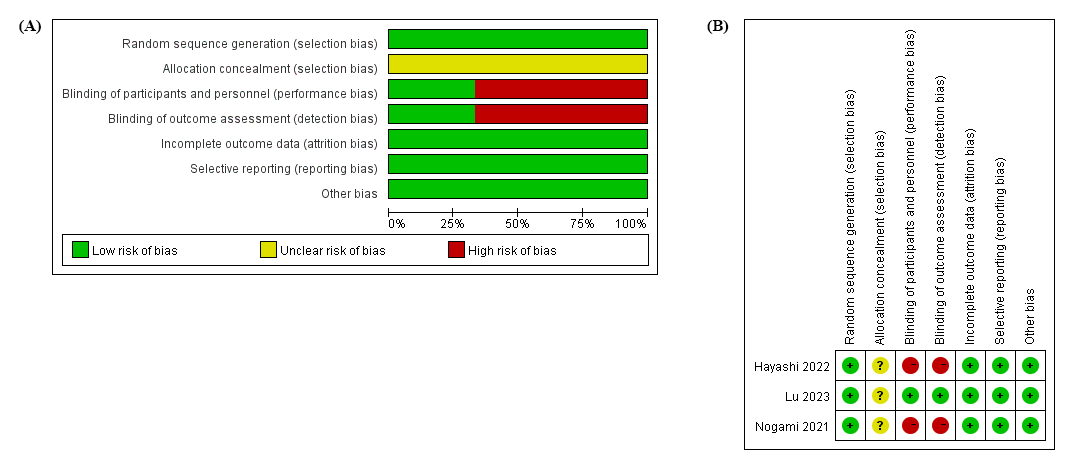

Supplement: Supplementary file 6 [file Image1.tif]

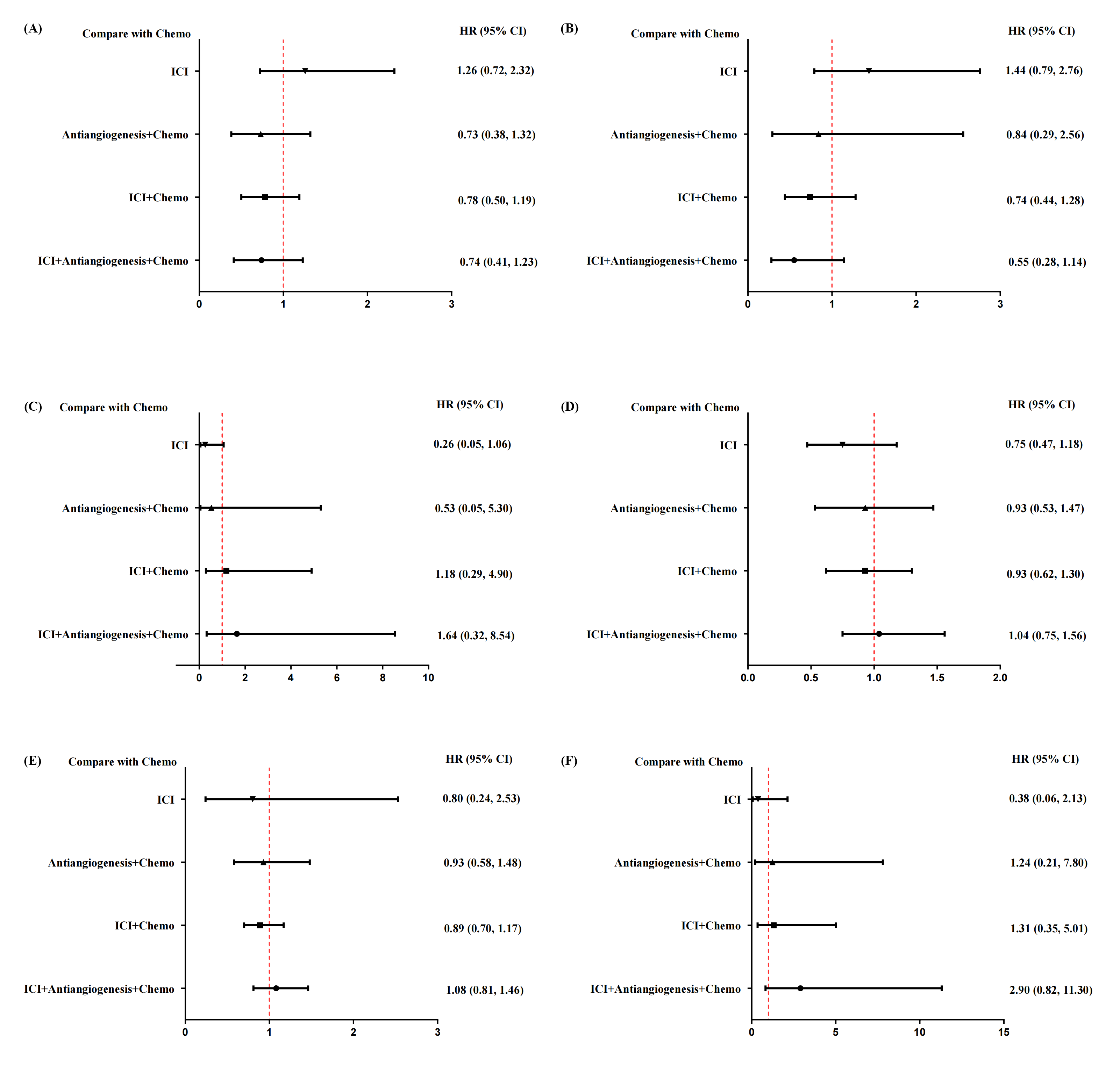

Supplement: Supplementary file 7 [file Image2.tif]

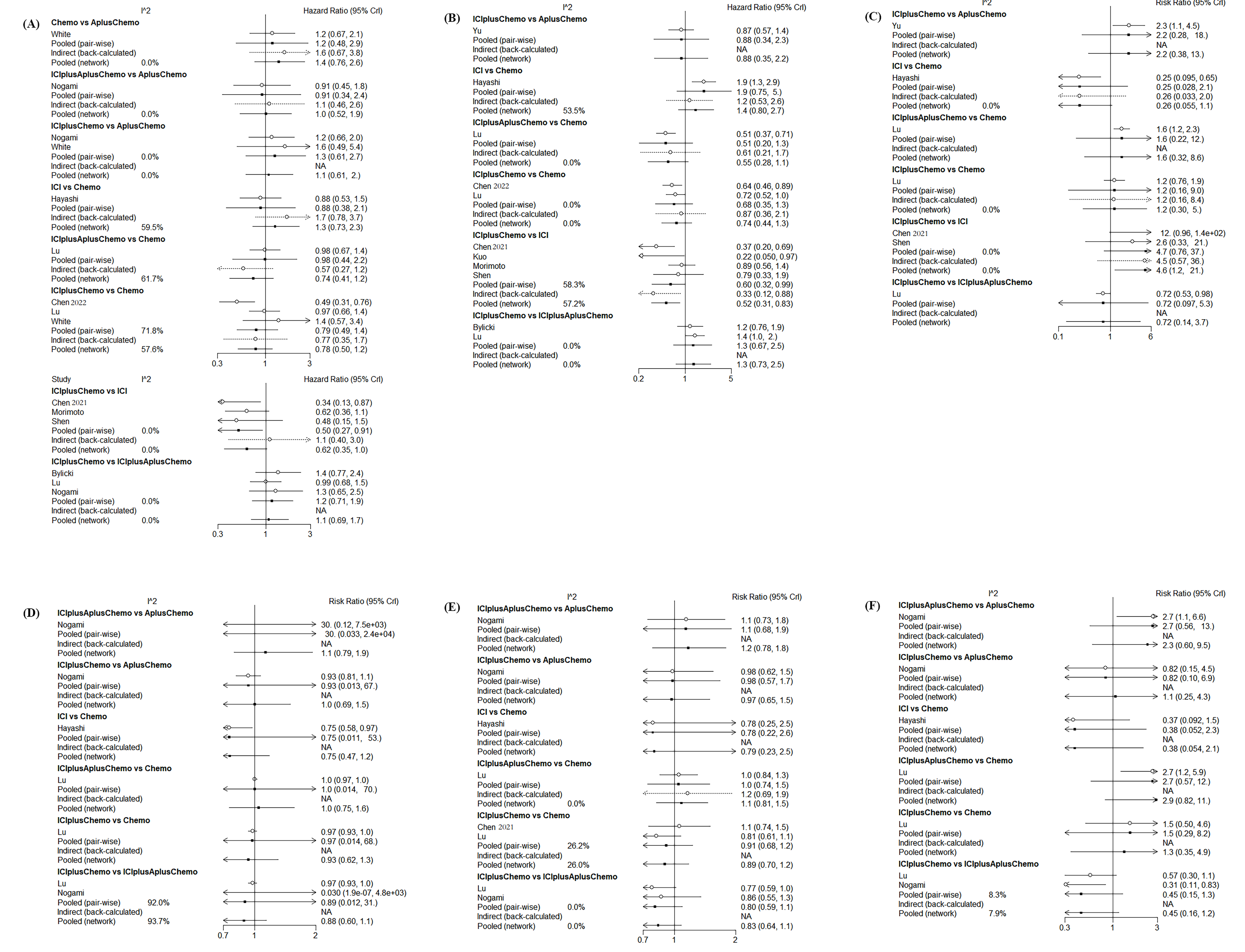

Supplement: Supplementary file 8 [file Image3.tif]

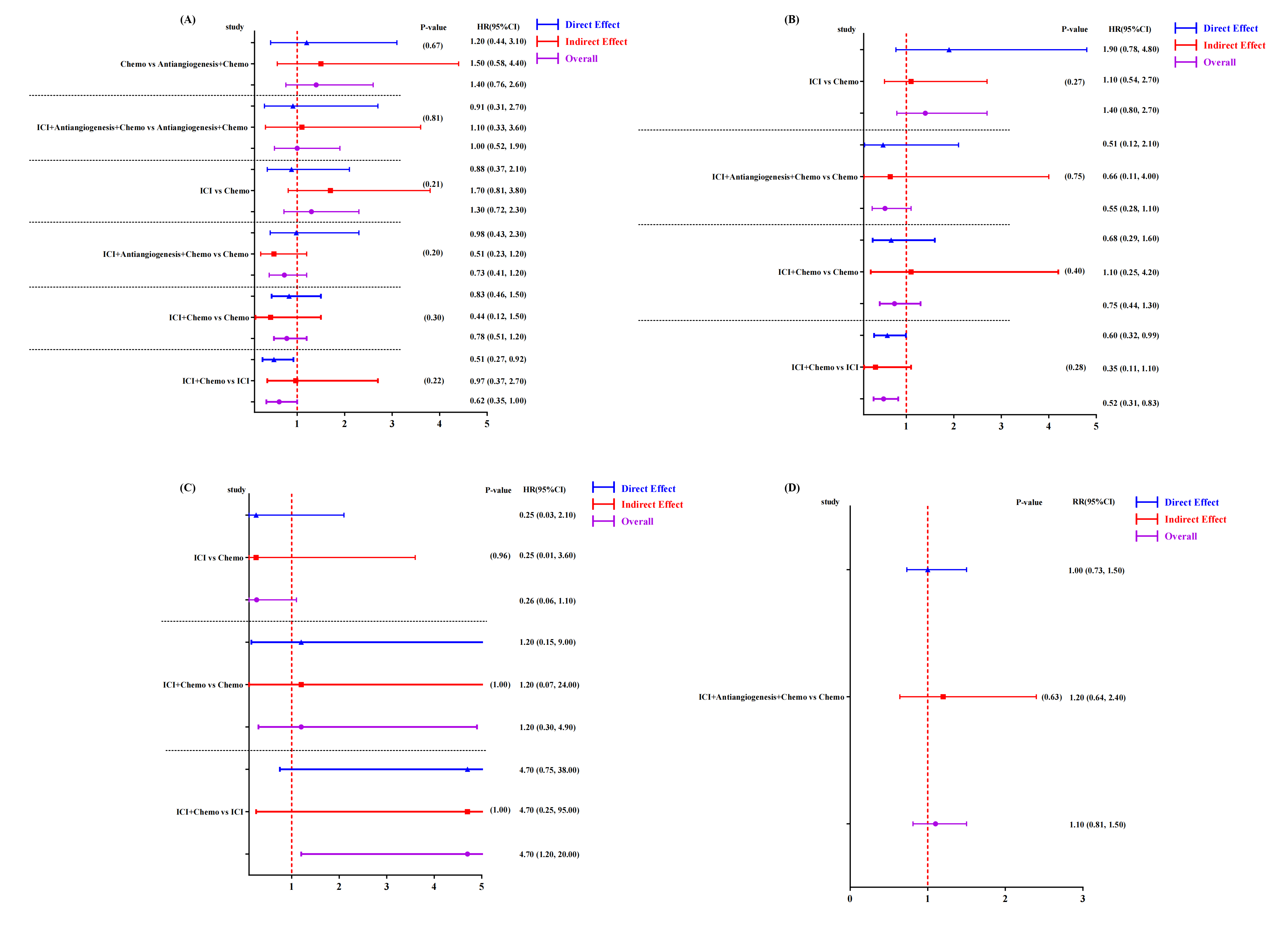

Supplement: Supplementary file 9 [file Image4.tif]

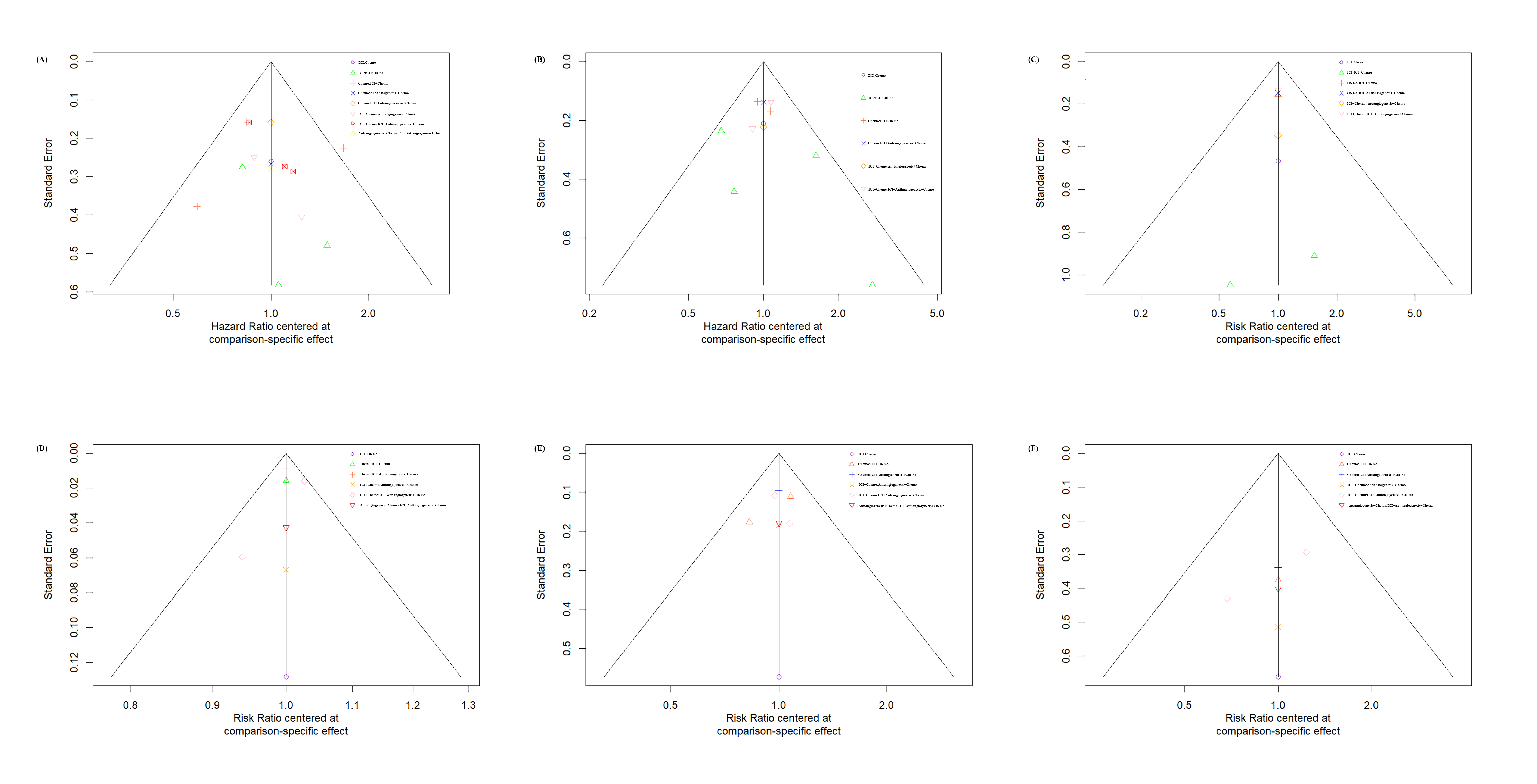

Supplement: Supplementary file 10 [file Image5.tif]
